# Practical Work in Medical Economics

**Published:** 1914-10

**Authors:** 


**A Monthly Review of Medicine and Surgery**

EDITOR AND PUBLISHER, DR. A. L. BENEDICT, 228 Summer St., corner of Elmwood Ave., Buffalo, (Address for all communications. Please make personal and telephone calls before 1 P. M.)

Third, in age, on the western continent. Independent, but supports professional organization. News limited mainly to Western N. Y. and Penn.

Subscription $2.00 a year; $3.00 for two years in advance. Changes and errors in address or failure or duplication of delivery, should be reported immediately.

Yearly Volume 70. OCTOBER, 1914 No. 3

### Practical Work in Medical Economics

It has been a source of pride to us that, from the time of our associate editorship on the defunct Medical and Surgical Reporter of Philadelphia, twenty years ago, we have reiterated the idea that the solution of the problems of medical economics involves the production of fewer physicians, and have, of course, pointed out that the only feasible way to accomplish this, was by the elevation of educational standards. We have also called attention to the fact that the same process would solve a good many ethical problems, not only along the lines of hospital, dispensary and college abuses for the obvious reason that a man with plenty of work to do is not apt to enter into unwholesome competition under the guise of charity and philanthropy, but in regard to quackery, fee splitting, etc., because good principles and kindness require in most persons, a fairly well filled stomach. We do not claim that the reiteration of these ideas was original. Many others said the same things. It would be equally absurd to claim any particular influence in accomplishing the really great things that have already been accomplished. But we did take pride in having expressed the right idea, clearly and on all proper occasions and thus in having contributed our share to the result.

But, recently, it occurred to us that we have done nothing but talk in regard to this matter. The writer went into medicine because he liked it, in spite of the reasons against such a course that he realized as a student and has subsequently expressed. He has assassinated none of the too numerous members of the profession by deed or word. He has, on several occasions declined a livelihood, sometimes strictly medical but outside of private practice, sometimes entirely non-medical.

It occurred to us, some time ago, on a day when we met four reformed physicians engaged in various business pursuits, that something practical might be done to relieve the over supply of physicians and to render it possible to carry out such slogans as 500,000 population, 500 physicians for Buffalo, 300,000 and 300 physicians for Rochester and so on for other cities and villages of Western N. Y. What we propose is this and it is only a humble effort in a great cause: If any medical graduate realizes that he is a unit in the over-supply, and wishes assistance either to find a situation in non-medical lines or in medicine outside of private practice or is willing to seek a new locality where there is a genuine vacancy and demand for a physician, we will respect his confidence, do everything in our power to assist him and invite from our medical readers, advertisers and others, information as to any such openings.

